# Concurrent genotyping of *Helicobacter pylori *virulence genes and human cytokine SNP sites using whole genome amplified DNA derived from minute amounts of gastric biopsy specimen DNA

**DOI:** 10.1186/1471-2180-8-175

**Published:** 2008-10-08

**Authors:** Anna Ryberg, Kurt Borch, Yi-Qian Sun, Hans-Jürg Monstein

**Affiliations:** 1 Divisions of Surgery, University Hospital, S-581 85 Linköping, Sweden; 2 Clinical Microbiology, University Hospital, S-581 85 Linköping, Sweden; 3 Department of Clinical and Experimental Medical, Faculty of Health Sciences, Linköping University, S-581 85 Linköping, Sweden

## Abstract

**Background:**

Bacterial and cellular genotyping is becoming increasingly important in the diagnosis of infectious diseases. However, difficulties in obtaining sufficient amount of bacterial and cellular DNA extracted from the same human biopsy specimens is often a limiting factor. In this study, total DNA (host and bacterial DNA) was isolated from minute amounts of gastric biopsy specimens and amplified by means of whole genome amplification using the multiple displacement amplification (MDA) technique. Subsequently, MDA-DNA was used for concurrent *Helicobacter pylori *and human host cellular DNA genotyping analysis using PCR-based methods.

**Results:**

Total DNA was isolated from gastric biopsy specimens of 12 subjects with gastritis and 16 control subjects having a normal mucosa. The DNA was amplified using a multiple displacement amplification (MDA) kit. Next, concurrent genotyping was performed using *H. pylori*-specific virulence gene PCR amplification assays, pyrosequencing of bacterial 16S rDNA and PCR characterisation of various host genes. This includes Interleukin 1-beta (*IL1B*) and Interferon-gamma receptor (*IFNGR1*) SNP analysis, and Interleukin-1 receptor antagonist (*IL1RN*) variable tandem repeats (VNTR) in intron 2. Finally, regions of the *vacA*-gene were PCR amplified using M13-sequence tagged primers which allowed for direct DNA sequencing, omitting cloning of PCR amplicons. *H. pylori *specific multiplex PCR assays revealed the presence of *H. pylori cagA *and *vacA *genotypic variations in 11 of 12 gastritis biopsy specimens. Using pyrosequencing, 16S rDNA variable V3 region signatures of *H. pylori *were found in 11 of 12 individuals with gastritis, but in none of the control subjects. Similarly, *IL1B *and *IFNGR1*-SNP and *IL1RN*-VNTR patterns could be established in all individuals. Furthermore, sequencing of M13-sequence tagged *vacA*-PCR amplicons revealed the presence of highly diverse *H. pylori vacA*-s/i/m regions.

**Conclusion:**

The PCR-based molecular typing methods applied, using MDA-amplified DNA derived from small amounts of gastric biopsy specimens, enabled a rapid and concurrent molecular analysis of bacterial and host genes in the same biopsy specimen. The principles and technologies used in this study could also be applied to any situation in which human host and microbial genes of interest in microbial-host interactions would need to be sequenced.

## Background

The Gram-negative spiral shaped bacterium *Helicobacter pylori *[1] is associated with the development of a variety of gastroduodenal diseases such as chronic gastritis, peptic ulcer disease and gastric cancer [2,3]. Virulence factors have been identified, including the production of urease (*ureA*) [4], a vacuolating cytotoxin (*vacA*) [5,6], and a cytotoxin-associated antigen (*cagA*) [7,8]. It has been implied that *ureA *[9], superoxide dismutase (*sod*) [10] and heat-shock protein 60 (*hsp60*) [11], found exclusively within the cytoplasm in other bacteria, are associated with the outer membrane in *H. pylori*. It is assumed that the variation in disease progression between patients is likely due to differences in bacterial virulence genes.

The *H. pylori *cytotoxin gene *vacA *is an important virulence marker. DNA sequence analysis has revealed that the *vacA *has a mosaic structure comprising allelic variations in the signal and midregion, each having two different alleles (s1/s2, m1/m2) with different biological activities [5,12]. Furthermore, it has been shown that the repeated hydrophilic motif region (RHM) is a potential proteolytic cleavage-site that separates the VacA-94 kDa protein into a 58-kDa and a 37-kDa protein with different biological activities [6,13]. Amino-acid substitutions, insertions and deletions within the RHM-region have been reported [14,15]. It has been speculated that mutations such as truncations, insertions and deletions in the midregion of the *vacA *gene which lead to in-frame stop codons, are associated with non-toxic *H. pylori *strains [14]. Recently, it has been shown that the *vacA *intermediate region (*vacA *i-region) may provide an important and independent marker of VacA-associated pathogenicity [16].

It is well recognised that the development of gastric disease is strongly influenced by host genetic factors. Cytokines play an important role in *H. pylori*-induced disease. An association of increased risk of gastric cancer and *IL1B *polymorphisms [17-19] and *IL1RN *polymorphisms [17,19-21] has been established. The *IFNGR1 *allele *2 has been associated with high *H. pylori*-reactive immunoglobulin G antibody concentrations [22] and an increased risk for atrophic gastritis [23]. Thus, for characterisation of bacterium-host interactions, it would appear that a concurrent typing of *Helicobacter*-specific virulence genes and cytokine polymorphisms in the same DNA, isolated only once from a biopsy specimen, is desirable.

Numerous PCR assays have been developed for the identification of *H. pylori*-specific virulence genes, reviewed in [15]. To reduce the number of *H. pylori*-specific virulence gene-based PCR amplification assays needed for such characterisation, we and others have developed multiplex PCR amplification assays which allow a highly sensitive detection and accurate genotyping of *H. pylori*-DNA [15,24-26]. A recent study described a method along these lines which allowed for concurrent genotyping analysis of *H. pylori *16S rDNA, 23S rDNA, *cagA *genes, and the cellular *IL1B *gene [27].

However, due to the low amount of bacterial DNA present in human biopsy specimens, the molecular identification of bacteria is often regarded impossible without culturing. To increase the specificity and sensitivity of the analysis of *H. py*lori infected biopsy specimens, nested PCR amplification in itself [28] or combined with Southern blot analysis of PCR amplicons has been used for molecular typing [29,30]. Identification of PCR amplicons by DNA sequence analysis employing time-consuming and expensive cloning, and subsequent plasmid DNA isolation procedures has also been used. However, these methods are not well suited for large-scale epidemiological screening and in this context, alternative techniques such as direct sequencing of M13-sequence tagged PCR amplicons for molecular typing of i. e. *Staphylococcus aureus *protein A (SPA-typing) [31], and whole genome amplification (WGA) of DNA using multiple displacement amplification (MDA) have been developed [32,33].

MDA is a PCR independent isothermal amplification technique that relies on the amplification of total genomic DNA, using a chemically modified random hexamer primer at 30°C and a bacteriophage Phi29 proofreading DNA polymerase, completing amplification within a few hours [32,34]. This technique dramatically improves the high-fidelity production of large amounts of genomic DNA with uniform coverage of genes [35-37]. We have recently reported on the use of MDA for amplification of DNA isolated from human intestinal biopsies [38], human gallstones specimens [39], and archival plasma/serum samples [40] for subsequent use in molecular typing of *H. pylori *and host genes.

In this study, we report on the use of MDA-amplified DNA for the molecular analysis of bacterial and human host genes in minute amounts of gastric biopsy specimens. Moreover, we report on the successful PCR amplicon sequencing, using a single, universal M13 uni (-21) primer targeting all M13-sequence tagged PCR amplicons.

## Methods

### Study subjects, tissue collection and DNA isolation

Fresh frozen (-80°C) archival tissue samples from 28 subjects from a previous study which was approved by the local ethics committee and conducted in accordance with the Helsinki Declaration, including informed written consent, were used [41]. Based on histological findings and rapid urease test from a larger (n = 501) gastroscopic screening study in a randomly selected cohort of the population of Linköping, Sweden [41], a total of 28 individuals (14 men; median age 58, range 42–73 and 14 women; median age 65, range 43–76) were selected and included in the present study. Among the 12 individuals with gastritis, four had antrum gastritis, five had pangastritis and two subjects had corpus gastritis with *H. pylori *infection, respectively, and one individual had corpus gastritis without an obvious *H. pylori *infection. All sixteen control subjects were *H. pylori*-negative and had a normal gastric mucosa (for details see table 1). DNA from gastric biopsy specimens and bacterial strains *Helicobacter pylori *26695 and J99, respectively, were extracted using a BioRobot M-48 Workstation and a MagAttract DNA Mini-48 kit as recommended by the manufacturer (Qiagen, Hilden, Germany).

**Table 1:** Sample information and *H. pylori *16S rDNA V3 sequence motif.

**Number**	**Gastritis classification^a^**	***H. pylori *16S rDNA^b^**
1	N	-
2	N	-
3	N	-
4	N	-
5	N	-
6	P-2-na	"strain A"^c^
7	N	-
8	C-1-na	-
9	A-1-a	J99
10	N	-
11	N	-
12	C-3-a	26695
13	N	-
14	A-1-a	26695
15	N	-
16	N	-
17	N	-
18	P-1-na	26695
19	N	-
20	N	-
21	P-1-na	J99
22	C-1-a	J99
23	P-2-na	26695
24	N	-
25	P-2-na	26695
26	N	-
27	A-1-a	26695
28	A-1-na	26695/J99^d^

Antral biopsy specimens, type of gastritis and *H. pylori *16S rDNA variable V3 region sequence motif derived from whole genomic amplified gastric biopsy DNA.

a) Gastritis classification according to Sydney system [57].

**N**: normal gastric mucosa; **A**: antrum predominant gastritis; **P**: pangastritis; **C**: corpus predominant gastritis. **1**: mild degree; **2**: moderate degree; **3**: severe degree. **a**: atrophy; **na**: no atrophy.

b) Based on pyrosequencing analysis.

c) The 16S rDNA variable V 3 region sequence motif corresponds to *H. pylori *"strain A", [GenBank:DQ059082] (Fig. 2, group III).

d) The 16S rDNA variable V 3 region sequence motif corresponds to a *H. pylori *26695/J99-like combination of *H. pylori *26695 and J99 (Fig. 2, group IV).

### Generation of MDA-amplified DNA

MDA of the isolated DNA was carried out using a GenomiPhi DNA amplification kit as recommended by the manufacturer (GE-Healthcare, Uppsala, Sweden). DNA concentrations were determined using a ND-1000 spectrophotometer (Nanodrop Technologies, Wilmington, DE, USA). Three separate MDA reactions were performed from each sample. The integrity of the MDA-amplified DNA was analysed by Ethidium bromide stained agarose-gel electrophoresis. Appropriate amounts (usually 1 to 2 μl) were used in subsequent PCR amplification assays.

### 18S rRNA and 16S rRNA PCR amplification

To evaluate the integrity of the prepared MDA-amplified DNA derived from total biopsy DNA, subsequent 18S rDNA and 16S rDNA PCR amplification was performed. In brief, 18S rDNA PCR amplification was carried out using a Quantum 18S rDNA internal standard kit as recommended by the manufacturer (Ambion, Austin, TX, USA) yielding a 489 bp PCR amplicon. 16S rDNA PCR amplification yielding an approximately 450 bp amplicon was carried out using 16S rDNA broad-range primers [42] flanking the variable V3, V4 and V9 regions (Table 2) and PCR amplification condition No 3 (Table 3) in a final 25 μl reaction volume using thin walled tubes, a HotStarTaq Master-mix kit (Qiagen, Hilden, Germany), and a PTC-100 Thermocycler (MJ-Research; SDS-Biosciences, Falkenberg, Sweden) or Mastercycler gradient (Eppendorf, Hamburg, Germany) as previously described [38].

**Table 2:** Oligonucleotides used in the present study.

**Primer**	**Primer sequence 5'-3' direction**	**PCR product (bp)**	**Gene/Region**	**Ref.^d^**
pJB-1.SE	attcgatgcaacgcgaagaaccttacc	~430	16S rDNA	
p13B.AS	gtgtactaggcccgggaacgtattc		(V3, V4, V9)	[38]
	
bio-HJ-HP-JBS.V3.SE	bio-cctaggcttgacattgaiagaa	~90	*HP*-16S rDNA	
B-V3.AS	acgacagccatgcagcacct		pyrosequencing	

*vacA*-1.SE	caatcgtgtgggttctggagc	678	*vacA*	
*vacA*-3.AS	gccgatatgcaaatgagccgc			[15]
	
*hsp60*-1.SE	gctccaagcatcaccaaagacg	603	*hsp60*	
*hsp60*-3.AS	gcggtttgccctctttcatgg			
	
*ureI*-3.SE	cagcaatgggatttgcgggttaacca	511	*ureI*	
*ureI*-4.AS	gatccaagcggttaaaataccctcaatgga			
	
*sod*-1.SE	gccctgtggcgtttgatttcc	425	*sodB*	
*sod*-3.AS	catgctcccacacatccacc			
	
*ureA*-1.SE	gcggctgaattgatgcaagaagg	350	*ureA*	
*ureA*-3M.SE	gctcgcaatgtctaagcgtttaccgaa			
	
*cagA*-2.SE	gaaatttggggatcagcgttacc	180	*cagA*	
*cagA*-3.AS	tcctgcaaaagattgtttggcaga			

VAI-F	atggaaatacaacaaacacac	259/286	*vacA *s1/s2	
VAI-R	ctgcttgaatgcgccaaac			[26]
	
VAG-F	caatctgtccaatcaagcgag	567/642	*vacA *m1/m2	
VAG-R	gcgtcaaaataattccaagg			

M13^a^-SeqS.SE	cgttgtaaaacgacggccagtga-ccctttgtgcaaaaatcgtt	381	vacA gene,	
SeqS.AS	cccarcctccatcaatctt		signal region	
	
M13^a^-SeqVac.SE	cgttgtaaaacgacggccagtga-gccaattcaayggcaattct	803	vacA RHM	pr.
SeqVac.AS	cgcttgattggacagattga		and i-region	
	
M13^a^-SeqM.SE	cgttgtaaaacgacggccagtga-agtcrttgatgggccttttg	717^b^	vacA gene, midregion	

IL1B-511F1.SE	tatgttctctgccccagcca	133	*IL1B*-511	
bio-IL1B-511R1.AS	bio-aatagccctccctgtctgtattga		rs16944^c^	pr.
Seq-IL1B-511.S1	gcaattgacagagagct		pyrosequencing	

bio-IL1B-31F1.SE	bio-atttctcagcctcctacttctgc	76	*IL1B*-31	
IL1B-31R1.AS	aagaggtttggtatctgccagttt		rs1143627^c^	pr.
Seq-IL1B-31.S1	ccctcgctgtttttat		pyrosequencing	

hIL1B+3954.AS	cggagcgtgcagttcagtgat	152	*IL1B *+3954	
bio-IL1B+3954.SE	bio-aattttgccgcctcgcctca		rs1143634^c^	[27]
IL1B+3954	cgttatcccatgtgtc		pyrosequencing	

IFNGR1.SE	ggtgacggaagtgacgtaagg	106	*IFNGR1*-56	
bio-IFNGR1.AS	gaggagagccatgctgctac		rs2234711^c^	pr.
Seq-IFNGR1.S2	gccggggctggaggg		pyrosequencing	

IL1RNF1.SE	cccctcagcaacactcc	270/356/442	IL1RN 86bp	[17]
IL1RNR1.AS	ggtcagaagggcagaga	/528/614	VNTR/Intron 2	

a) underlined sequence corresponds to a M13-sequence adapter used in DNA sequencing.

b) expected PCR product using M13-SeqM.SE and VAG-R primers.

c) rs: Reference SNP identification number [58]

d) pr. – present study.

**Table 3:** PCR amplification conditions used in the present study.

**PCR No**	**Initial denaturation**	**Denaturation**	**Annealing**	**Extension**	**No of cycles**	**Final extension**
1	95°C; 15 min	95°C; 30 s	56°C; 30 s	72°C; 1 min	30	72°C; 10 min

2	95°C; 15 min	95°C; 30 s	62°C; 30 s	72°C; 1 min	30	72°C; 10 min

3	95°C; 15 min	95°C; 30 s	55°C; 30 s	72°C; 1 min	30	72°C; 10 min

4	95°C; 15 min	95°C; 30 s	57°C; 30 s	72°C; 1 min	37	72°C; 10 min

5	95°C; 15 min	95°C; 30 s	65°C, 30 s	72°C; 30 s	5	72°C; 10 min
		95°C; 30 s	60°C; 30 s	72°C; 30 s	25	
		95°C; 30 s	55°C; 30 s	72°C; 30 s	5	

### Pyrosequencing analysis

Pyrosequencing template generation was performed using 16S rDNA broad-range PCR amplicons as a template (see above). For that purpose, broad-range 16S rDNA PCR amplicons were purified using a GFX PCR DNA and Gel-band purification kit (GE Healthcare, Uppsala, Sweden) and pyrosequencing PCR amplicons were generated using an appropriate amount (usually 1 μl) of 16S rDNA template, a HotStarTaq Master mix kit (Qiagen AB, Solna, Sweden), 5 pmol each of *Helicobacter*-specific primer 5'-biotin HJ-HP-JBS.V3 and broad-range primer B-V3.AS (Table 2), both flanking the 16S rDNA V3 region only, and PCR condition No 1 (Table 3). Pyrosequencing analysis was carried out as described elsewhere [43] using a PSQ 96 MA System (Biotage AB, Uppsala, Sweden) and sequencing primer B-V3.AS (Table 2).

### *H. pylori*-specific virulence-gene multiplex PCR amplification

**Figure 1:**
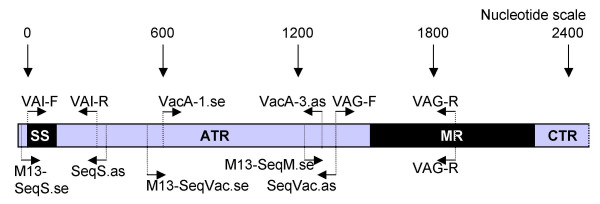
***VacA*-primers**. Schematic representation of the approximate location of the *vacA*-primers used in the present study (Table 2). SS = signal sequence region; ATR = amino-terminal region; MR = middle-region; CTR = Carboxyterminal region (nomenclature adapted from [50]).

*vacA*, *hsp60*, *ureI*, *sod*, *ureA *and *cagA *(Table 2) multiplex PCR amplification was carried out using 5 pmol of each primer and PCR amplification condition No1 (Table 3) [15]. For *vacA *subtyping, a duplex PCR using primers VAI-F and VAI-R specific for *vacA *s1/s2 genotypes, primers VAG-F and VAG-R (Fig. 1; Table 2) specific for *vacA *m1/m2 genotypes [24,26], and PCR condition No 1 were used (Table 3). PCR amplicons were analysed on the automated capillary electrophoresis QIAxcel system and a QIAxcel DNA high resolution kit (Qiagen, Hilden, Germany).

### Cytokine polymorphism analysis

MDA-amplified DNA was analysed by pyrosequencing for the presence of *IL1B*- and *IFNGR1*-polymorphisms. Primers flanking the *IL1B*-511 [ref SNP ID:rs16944],*IL1B*-31 [rs1143627]and *IFNGR1*-56 [rs2234711] regions were designed based on the available genomic *IL1B *[GenBank:AY137079] and *IFNGR1 *sequences [GenBank:AY594694] using a PSQ Assay Design software (Biotage AB, Uppsala, Sweden). Templates for pyrosequencing SNP analysis were obtained by PCR amplification using 1 μl of MDA-amplified DNA, 5 pmol of each primer flanking the *IL1B*-511, *IL1B*-31, *IL1B*+3954 [rs1143634] and the *IFNGR1*-56 regions (Table 2), and PCR amplification condition No 2 for *IL1B*-511, *IL1B*-31 and *IFNGR1 *primers and No 3 for *IL1B *+3954 primers (Table 3). Pyrosequencing analysis was performed as described elsewhere [40,43] with the modification of using a PSQ 96MA System, and the pyrosequencing primers specified in table 2.

The 86 bp variable number tandem repeat (VNTR) in intron 2 of the *IL1RN *gene was amplified using the primers IL1RNF1.SE and IL1RNR1.AS (Table 2) and PCR amplification No 5 (Table 3) followed by automatic capillary electrophoresis analysis using a QIAxcel system and a QIAxcel DNA high resolution kit (Qiagen, Hilden, Germany). So far, five VNTR alleles have been reported; allele *1–*5 having four, two, five, three, and six repeats, corresponding to amplicon sizes of 442 bp, 270 bp, 528 bp, 356 bp and 614 bp, respectively.

### DNA sequence analysis of *vacA *PCR amplicons

Sequence data from *Helicobacter pylori *J99 [GenBank:AE001439], *H. pylori *26695 [GenBank:AE000511], *H. pylori *NCTC 11638 [GenBank:U07145], and *H. pylori *unspecified [GenBank:U29401] for *vacA *primer design were retrieved from GenBank [44] and aligned using ClustalX [45]. Primers were designed and checked using Primer3 [46] and optimized by annealing temperature gradient PCR and Mg^2+ ^concentration.

To identify and establish *vacA *s/i/m genotype, 2 μl MDA-amplified DNA derived from the 11 *H. pylori*-positive biopsies were PCR amplified with three new *vacA*-specific primer pairs, using 10 pmol of each primer and PCR condition No 4 (Table 3). The s-region was amplified with primer M13-SeqS.SE and SeqS.AS, the RHM and i-region with primers M13-seqVac.SE and seqVac.AS, and the m-region with primers M13-seqM.SE and VAG-R (Fig. 1; Table 2) yielding M13-sequence (M13 uni, -21) tagged PCR amplicons. PCR amplicons were treated with ExoSAP-IT to inactivate excess of oligonucleotide primers, following the manufacturer's instructions (USB Europe GmbH, Staufen, Germany), and lyophilised. Subsequent DNA sequence analysis was carried out using a custom DNA sequencing service (Eurofins MWG GmbH, Martinsried, Germany). *H. pylori *MDA-amplified DNA No 18 was further amplified using the primer combination M13-SeqVac.SE and VAG-R at the conditions described above. The obtained sequences corresponding to the 11 *H. pylori*-DNA *vacA *s-regions were aligned and compared with catalogued *H. pylori vacA *type s1a [GenBank:AY185128], s1b [GenBank:AB057223], s2 [GenBank:AY438687], and s1c [GenBank:AB057107] sequences using CLC DNA workbench 3 (CLC bio A/S, Aarhus, Denmark). Similarly, DNA sequences corresponding to the *vacA *i/m-regions were aligned and compared with catalogued *H. pylori vacA *type i1/m1 [GenBank:U05676] and type i2/m2 [GenBank:U29401] sequences.

## Results

### MDA-amplified DNA

Each biopsy yielded approximately 2–4 μg total DNA (A_260/280 _> 1.8) in a final volume of 150 μl water. On average, 1 μl genomic DNA generated approximately 12–22 μg MDA-amplified DNA. 18S rDNA and 16S rDNA PCR amplification using MDA-amplified DNA yielded PCR amplicons of the expected sizes, 490 bp and 450 bp, respectively (data not shown).

### Presence of *Helicobacter *spp.-DNA in antrum biopsies

**Figure 2:**
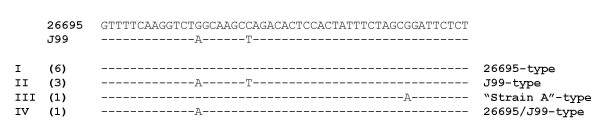
***H. pylori *16S V3 pyrosequencing results**. DNA sequence alignment of the 16S rDNA variable V3 region of *Helicobacter *isolates derived from pyrosequencing analysis. Dashes indicate sequence identity with *H. pylori *26695. Roman numerals indicate four *Helicobacter*-specific 16S rDNA variable V3 region sequence motif-groups [43]. The number of *Helicobacter *isolates in each group is indicated in parentheses. Group I, *H. pylori *strain 26695-like [GenBank:AE000620]: No 12, 14, 18, 23, 25, 27. Group II, *H. pylori *strain J99-like [GenBank:NC_000921]: No 9, 21, 22. Group III *H. pylori *strain A-like [GenBank:DQ059082]: No 6. Group IV, H. pylori 26695/J99-like: No 28 (see also table 1).

MDA-amplified DNA derived from 28 antrum biopsies was analysed for the presence of *Helicobacter *spp.-DNA. Pyrosequencing analysis revealed that 11 of 28 subjects were infected with *H. pylori. H. pylori *16S rDNA sequences were detected only in biopsy specimens from patients with diagnosed gastritis (Table 1). 16S rDNA variable V3 region sequence motifs corresponding to *H. pylori *26695 and J99 were detected in six and three samples, respectively. Two specimens had deviating V3 region sequence motifs (Fig. 2); *H. pylori *sequence No 6 corresponds to the V3 sequence motifs of *H. pylori *"strain A" [GenBank:DQ059082] and *H. pylori *sequence No 28 corresponds to a *H. pylori *26695/J99-like combination of 26695 and J99. In the remaining 17 biopsies, *Helicobacter *spp.-DNA was not detected (Table 1).

### Multiplex PCR amplification

**Figure 3:**
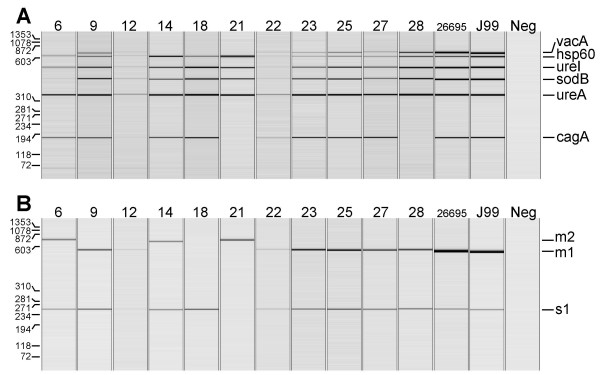
***H. pylori *multiplex PCR and *vacA *subtyping results**. Results from the automatic capillary electrophoresis of multiplex PCR amplicons derived from 11 *H. pylori*-infected subjects and control strains *H. pylori *26695 and J99. The positions of A)*H. pylori*-specific *vacA, hspA, ureI, sodB, ureA*, and *cagA *multiplex PCR amplicons and of B)*vacA *s1, *vacA *m1 and *vacA *m2 PCR amplicons are indicated. *Neg *represents a non-template control. A virtual internal reference marker is indicated in the left margin.

*H. pylori*-positive MDA-amplified DNA (based on 16S rDNA pyrosequencing analysis), and *H. pylori *26695 and J99-DNA as references (Table 1) were further analysed by the *H. pylori *virulence gene-based multiplex PCR amplification assay. Capillary electrophoresis revealed *hsp60, ureI, sodB*, and *ureA *PCR amplicons of the expected sizes in all MDA-amplified DNA derived from the *H. pylori *positive biopsies and the control strains. However, multiplex PCR amplification generated only weak bands in No 12 and 22, indicating the presence of a low level of *H. pylori *DNA in these biopsies (Fig. 3a). The *cagA*+/*vacA*+ combination was present in six MDA-amplified DNA samples (No 9, 12, 22, 23, 25, 27) and was also found in the control strains. The *cagA*+/*vacA*- combination was present in three biopsies (No 6, 14, 18), and the *vacA*-/*cagA*- and *vacA*+/*cagA*- combinations were found in one biopsy each (No 21 and 28, respectively; Fig. 3a).

### *VacA *subtyping by duplex PCR amplification

Multiplex PCR amplification of the 11 *H. pylori*-positive MDA-amplified DNA samples yielded seven PCR amplicons with a *vacA*+ genotype (Fig. 3a). We also used another *vacA*-specific duplex PCR amplification assay which allows for the discrimination between signal region s1 and s2 alleles, and midregion m1 and m2 alleles, generating PCR amplicons of distinct sizes (Table 2). *VacA *s/m-profiles were generated from all *H. pylori *positive MDA-amplified DNA (Fig. 3b). The *vacA *type s1/m1 was observed in seven MDA-amplified DNA samples (No 9, 12, 22, 23, 25, 27, 28) and the two control strains, and the *vacA *type s1/m2 was observed in two biopsies (No 6 and 14). In one case each, only a single band corresponding to a *vacA *type s1 (No 18) or a *vacA *type m2 (No 21) was found (Fig. 3b; Table 4).

**Table 4:** *H. pylori *genotyping results.

No	16S rDNA type^a^	MP-PCR		*vacA*-PCR^b^	DNA sequencing analysis^c^		
		*cagA*	*vacA*	*subtype*	*s-region*	*i-region*	*m-region*
6	"Stain A"	+	-	s1/m2	s1a	i2	m2
9	J99	+	+	s1/m1	s1a	i1	m1
12	26695	+	+	s1/m1	s1a	i1	m1
14	26695	+	-	s1/m2	s1a	i1-2^d^	m2
18	26695	+	-	s1/m?	s1a	i1-2^d^	m2
21	J99	-	-	s?/m2	s2	i2	m2
22	J99	+	+	s1/m1	s1b	i1	m1
23	26695	+	+	s1/m1	s1a	i1	m1
25	26695	+	+	s1/m1	s1a	i1	m1
27	26695	+	+	s1/m1	s1a	i1	m1
28	26695/J99	-	+	s1/m1	s1b	i1	m1
control	HP 26695	+	+	s1/m1	s1a	i1	m1
control	HP J99	+	+	s1/m1	s1b	i1	m1

*H. pylori *virulence-gene typing using MDA-amplified DNA. *H. pylori *26695 and J99 were included as control strains.

a) For details see table 1.

b) Question mark indicates the absence of a detectable PCR amplicon (s/m-genotype).

c) The s/i/m-region sequences were aligned and compared with sequences retrieved from GenBank (for details see Materials and methods).

d) The intermediate region represents a mixture of i1/i2 sequences.

### Cytokine SNP and IL1RN-VNTR analysis

MDA-amplified DNA derived from the 28 biopsies was used for pyrosequencing analysis of the human *IL1B-511*, *IL1B-31*, *IL1B+3954 *and *IFNGR-56 *SNPs (Table 5). In the 11 *H. pylori*-infected biopsy specimens, the SNP CC/CT/TT-genotype distribution was 4/5/2 for *ILB1*-511, 3/4/4 for *IL1B*-31, 8/3/0 for *IL1B*+3954, and 2/6/3 for *IFNGR1*-56. In the 17 *H. pylori*-negative biopsy specimens, the SNP CC/CT/TT-genotype distribution was 9/5/3 for *IL1B*-511, 3/5/9 for *IL1B*-31, 12/4/1 for *IL1B *+3954, and 2/6/9 for *IFNGR1*-56 (Table 5).

**Table 5:** Cytokine genotyping using MDA-amplified DNA.

Number	*IL1B*-511 (C/T)	*IL1B*-31 (T/C)	*IL1B +*3954 (C/T)	*IFNGR1*-56 (T/C)	*IL-1RN *86 bp VNTR
6*	T/T	C/C	C/C	T/T	1/2
9*	C/C	T/T	C/C	T/C	1/1
12*	C/C	T/T	C/T	T/T	1/2
14*	C/T	T/C	C/T	T/C	1/2
18*	C/T	T/C	C/T	T/C	1/2
21*	C/T	T/C	C/C	T/C	1/1
22*	C/T	T/C	C/C	C/C	1/1
23*	C/T	C/C	C/C	T/T	2/2
25*	C/C	T/T	C/C	C/C	1/1
27*	T/T	C/C	C/C	T/C	1/1
28*	C/C	T/T	C/C	T/C	1/1
1	C/T	T/C	C/C	T/C	2/2
2	C/C	T/T	C/T	T/T	1/1
3	C/C	T/T	C/T	T/T	1/1
4	C/C	T/T	C/C	T/T	1/1
5	C/T	T/C	C/C	T/T	1/1
7	C/C	T/T	C/C	T/T	1/1
8	C/T	T/C	C/C	T/T	1/1
10	C/C	T/T	C/C	T/C	1/3
11	T/T	C/C	C/C	T/C	1/2
13	C/T	T/C	C/T	T/C	1/2
15	C/C	T/T	T/T	T/T	1/1
16	T/T	C/C	C/C	T/T	2/2
17	C/C	T/T	C/C	T/C	2/2
19	C/C	T/T	C/T	T/C	1/1
20	C/C	T/T	C/C	C/C	1/2
24	C/T	T/C	C/C	C/C	1/1
26	T/T	C/C	C/C	T/T	1/2

* Indicates *H. pylori *positive individuals.

**Figure 4:**
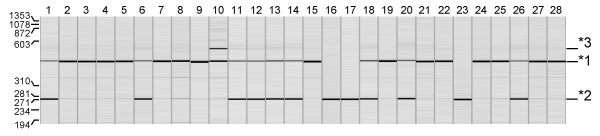
***IL1RN*-VNTR genotyping results**. Automatic capillary electrophoresis of *IL1RN*-VNTR PCR amplicons from all 28 biopsies. The number of each lane corresponds to the sample listed in table 1. The position of the virtual, internal marker and expected position of allele *1, *2 and *3 are indicated.

Capillary electrophoresis revealed an *IL1RN*-VNTR 1/1-genotype in six (No 9, 21, 22, 25, 27, 28), a 1/2-genotype in four (No 6, 12, 14, and 18), and a 2/2-genotype in one (No 23) of the *H. pylori*-infected gastritis biopsies. The biopsy specimen having gastritis without *H. pylori *infection (No 8) revealed an *IL1RN*-VNTR 2/2-genotype. An *IL1RN*-VNTR 1/1-genotype was found in eight (No 2, 3, 4, 5, and 7), a 1/2-genotype in four (No 11, 13, 20, 26), a 1/3-genotype in one (No 10), and a 2/2-genotype in three (No 1, 16, 17) of the histological normal biopsies (Fig. 4; Table 5).

### *VacA *subtyping by DNA sequence analysis

**Figure 5:**
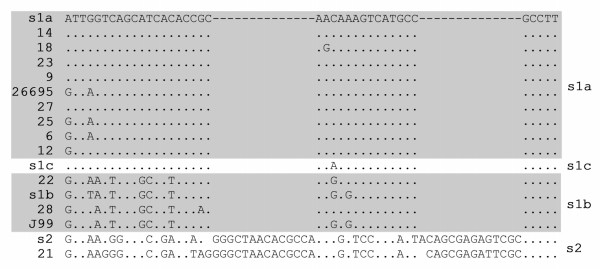
***VacA *s-region nucleotide sequence alignment**. DNA sequence alignment of obtained *vacA *s-region. The *vacA *type s1a and s1b sequences are shaded in grey. Reference sequences are indicated with NCBI accession number [GenBank:AY185128] – s1a, [GenBank:AB057107] – s1c, [GenBank:AB057223] – s1b, and [GenBank:AY438687] – s2. Dots indicate nucleotide sequence identical to the one above and gaps indicate deletions. The gaps in the reference sequence are indicated by dashes.

**Figure 6:**
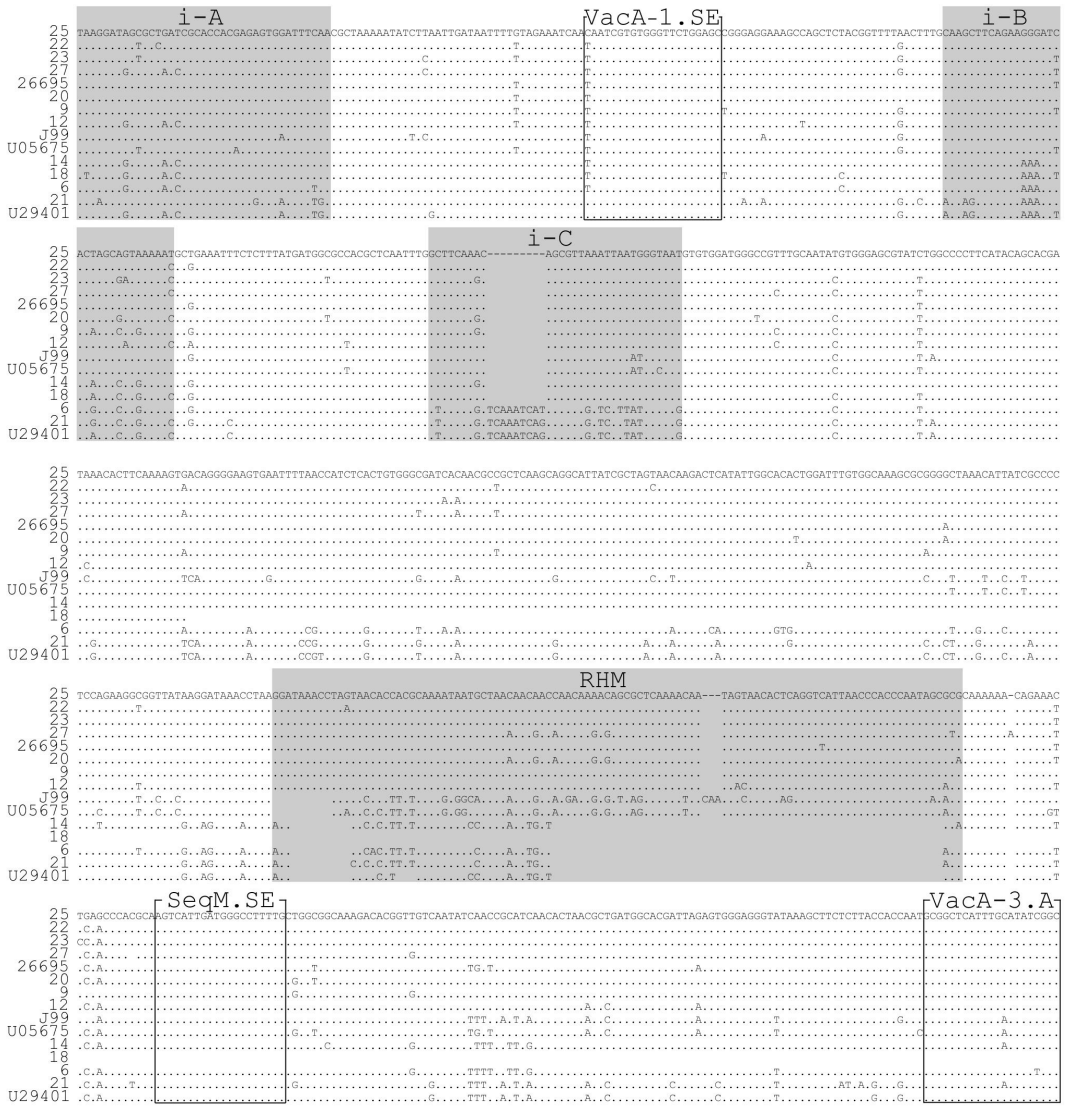
***VacA *i- and RHM-region nucleotide sequence alignment**. DNA sequence alignment of the obtained sequences containing intermediate A, B and C, and RHM regions, which are shaded in grey. Reference sequence [GenBank:U05675] (i1) and [GenBank:U29401] (i2) are obtained from Rhead et al. [16]. Dots indicate identical nucleotide sequences and gaps indicate nucleotide deletions. Primer binding sites are indicated with boxes.

**Figure 7:**
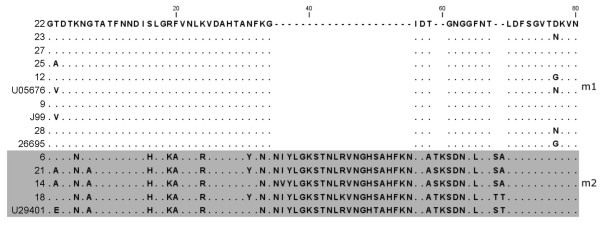
***VacA *m-region protein sequence alignment**. Alignment of deduced amino acid sequence, translated from the obtained *vacA *m-region DNA sequences. Samples with m2 genotype are shaded in grey. Reference sequence [GenBank:U05675] (m1) and [GenBank:U29401] (m2) are obtained from Rhead et al. [16]. Dots indicate identical nucleotide sequences and gaps indicate nucleotide deletions.

**Figure 8:**
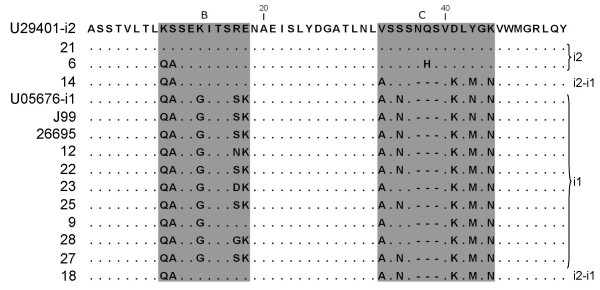
***VacA *i-region protein sequence alignment**. Amino acid sequence alignment from the *vacA *variable i-regions. Shaded areas indicate the important B and C regions described by Rhead et al. [16].

DNA sequencing analysis revealed the presence of different *vacA *genotypes in all *H. pylori *strains, indicating that sequencing of M13-sequence tagged PCR amplicons is a more discriminating molecular typing approach than analysis of PCR amplicons by capillary gel electrophoresis as done in this study (Fig. 5, 6, 7, 8; Table 4). The apparent absence of *vacA *amplicons in the multiplex PCR analysis of sample no 6, 14, 18 and 21, is explained by a 70 bp deletion in the RHM region (Fig. 6). The PCR products for these samples are approximately 600 bp and coincide with the *hsp60 *amplicon of similar size (Fig. 3a; Table 2).

DNA sequence alignment revealed ten *vacA *s1a genotypes (No 6, 9, 12, 14, 18, 23, 25, 27 and reference strain 26695), three s1b genotypes (No 22, 28, and strain J99) and one s2 genotype (No 21) when compared to *H. pylori *s1a [GenBank:AY185128], *H. pylori *s1b [GenBank:AB057223], and *H. pylori *s2 [GenBank:AY438687] as reference sequences (Fig. 5).

Nine *H. pylori *strains had a *vacA *type m1 (No 9, 12, 22, 23, 25, 27, 28, and reference strains 26695 and J99) and four had a type m2 (No 6 14, 18, 21) (Fig. 7; Table 4). Both i1 and i2 types, but also several chimeric types were identified. All the m1-type strains revealed an i1 genotype, No 21 (s2m2) had an i2, and the s1m2-strains (No 6, 14, and 18) had a chimeric i-region structure (Fig. 8).

DNA sequence analysis of a 900 bp PCR amplicon, produced using MDA-amplified DNA from biopsy No 18 and primers M13-seqVac.AS and VAG-R (Fig. 1), revealed a large deletion in the midregion of the *vacA *gene resulting in the absence of several primer target sites (Fig. 6). This explains the absence of a *vacA *m1/m2 genotype in the *vacA*-subtyping PCR amplification assay of biopsy No 18 (Fig. 3b).

## Discussion

Rapidly and easily acquired information on bacterial and host genes is becoming increasingly important for diagnosis and decision-making when choosing suitable therapies for human infectious diseases. This makes new demands on clinical research and routine laboratories to develop molecularly based methods that can accurately identify and characterise bacterial virulence and host susceptibility and/or resistance genes. The amount of genomic DNA available for such genetic analyses is often limited. Quite often, only minute amounts of bacterial DNA can be found in human biopsies. Moreover, it is desirable to perform concurrent, multiple bacterial and host genotyping analyses from the same, limited amount of biopsy DNA. In such studies, the traditional approach is to purify DNA from cultured bacterial strains isolated from the biopsy specimens [27]. Sample preparation is commonly carried out using time-consuming cartridge or bead-based techniques. These methods do not allow an efficient high-throughput analysis of clinical samples and significant variations of DNA yield and purity can be observed. By contrast, an automated nucleic acid extractor combined with MDA-amplification yields DNA of high purity and integrity that can be used in downstream applications. Indeed, recent studies have demonstrated that PCR amplifications using MDA-amplified DNA can also be carried out under conditions where PCR amplifications normally are hampered due to the presence of PCR inhibitors [47].

In recent years, MDA has been tried out for the amplification of microbial DNA [36,48,49] and total DNA (bacterial and cellular) isolated from human biopsy specimens [38,39]. This study shows the feasibility of using MDA-amplified total DNA, isolated from human biopsy specimens, for research and clinical diagnostic analysis of both host and infecting bacterium in the same DNA pool.

Pyrosequencing analysis of the16S rDNA variable V3 region revealed the presence of different *H. pylori *subspecies in the different biopsy specimens which is in agreement with previous reports showing that subtle DNA sequence variations occur in the 16S rDNA variable V3 region of *H. pylori*, providing a consistent system for subtyping [30,43]. The taxonomy of these *H. pylori *strains may be a matter of debate. Subdivision of the species *H. pylori *into subspecies, based on biotypes, pathotypes or serotypes for taxonomic as well as clinical reasons has been suggested [50,51].

Pro-inflammatory *IL1B *and *IL1RN *polymorphisms are associated with increased risk of gastric carcinoma in Caucasian populations [52]. Carriers of these pro-inflammatory polymorphisms revealed an increased *IL1B *gene expression pattern [19,53] in the mucosa and increased prevalence of intestinal metaplasia and atrophic gastritis [19]. Similarly, a genome-wide linkage analysis identified SNPs in *IFNGR1 *affecting *H. pylori *infection [22]. Due to the limited number of biopsies analysed, we were not able to draw any statistically significant conclusion regarding allele frequencies in *H. pylori*-infected and histologically normal individuals. However, the primary goal of the present study was not to perform a clinical study at large but rather to establish new methodological approaches. In analogy with these findings we have recently shown that MDA-DNA derived from minute amounts of archival plasma/serum DNA allowed us to identify cytokine polymorphic SNP-sites by means of pyrosequencing analysis [40]. The use of IL1B-SNP analysis by means of PCR-restriction-fragment-length polymorphism where IL1B-511, IL1B-31 and IL1B+3954 PCR amplicons are digested with restriction enzyme *Ava*I, *Taq*I and *Alu*I, respectively, is a widely accepted approach [19-21]. However, restriction enzyme digestion, followed by agarose gelelectrophoresis is a time consuming and, in a clinical routine laboratory context, a tedious process requiring up to 100 ng DNA in each assay [20]. Thus, we conclude that MDA-DNA derived from human biopsy specimens provides a reliable source for cytokine-SNP analysis and, therefore, the same approach may be applied for the characterisation of other host genetic factors in population studies at large

Incorporation of universal M13-sequencetags at the 5'-end of PCR primers facilitated straightforward sequencing of amplicons, which makes culturing of bacteria from human biopsy specimens and cloning of PCR amplicons prior to DNA sequencing unnecessary. This makes it possible for clinical routine laboratories using this technique to rapidly produce sequencing results. Sequencing of M13-tagged amplicons was first described for SPA-typing [31]. The technique has successfully been applied in our laboratory for direct sequencing of PCR amplicons in gene expression studies of vasopressin receptor mRNA splice-variants expressed in the human gastro-intestinal tract and surrounding tissues [54]. In the present study we have used M13 sequence-tags for successful partial sequencing of the *H. pylori *virulence *vacA *gene (Fig. 6). Multiple *H. pylori *strain infection was not seen in this study, although the number of samples included is small. Primer design is of utter importance since species-specific primers are essential to gain pure and reliable sequencing results directly from DNA isolated from a mixed flora.

According to a recent study, the intermediate region (i-region) of the *H. pylori vacA *gene is an important and independent marker of VacA-associated pathogenicity [16]. All i1-type but no i2-type strains induce vacuolation. Chimeric versions of the i-region (i1-i2) can induce low level of vacuolation *in vitro*. According to this criteria, our results show two tentatively non-toxic strains (s2/i2/m2- No 21 and s1/i2/m2- No 6) and one with reduced toxicity (s1/i1-i2/m2 No 14; No 18 is discussed below). The remaining biopsies contain toxic *H. pylori *s1/i1/m1 strains (Table 4).

False negative PCR results from the multiplex-PCR (Table 4) were revealed when using new primers, targeting conserved regions of the *vacA *gene. The negative results were caused by deletions in the RHM region, yielding a smaller amplicon than expected by previous analysis (Fig. 6). One sample (No 18) had large deletions in the RHM region of the gene, leaving out *vacA*-primer binding sites commonly used in vacA PCR amplification assays (Fig. 1 and 6). The sequence contained several premature stop codons downstream of the deletion (data not shown), indicating a possible inactive form of VacA. However, further analysis is needed in order to establish if this strain can produce an active protein.

The conclusions to be drawn from the present data is that the choice of primers in *vacA *PCR amplification assays influences the apparent prevalence *vacA*-positive *H. pylori *strains and, hence, precaution has to be taken in the interpretation of *vacA*-negative PCR amplification results. Thus, our results are in good agreement with previous reports that revealed a high level of *vacA *genotype variation such as single nucleotide mutations, deletion and in frame stop codons in the *vacA *alleles of non-toxic *H. pylori *strains [14,15], but it seems questionable to what extent published *vacA *PCR amplification results can be compared between studies if DNA sequencing is not performed. From our and other studies it seems evident that it is necessary to establish partial DNA sequences from either *vacA *PCR amplicons (Fig. 3, 5, 6) or from full-length *vacA *open reading frames. Thus, high-throughput sequencing of M13 sequence-tagged PCR amplicons appears to be a more promising approach, both in research and clinical routine laboratories. This principle could be applied to any situation in which sequencing of PCR amplicons is desirable and thereby, one could abstain from tedious amplicon cloning and plasmid preparation procedures prior to DNA sequence analysis.

Histology is considered a sensitive method for detection of *H. pylori *in gastric biopsies. However, a recent study has demonstrated that PCR amplification using *H. pylori*-specific primers detected *H. pylori*-DNA in histological-negative gastric biopsies, indicating the clinical relevance of *H. pylori *detection by PCR amplification in biopsies with characteristic inflammatory changes [55]. Similarly, Kisa and co-workers [56] evaluated different diagnostic methods for the detection of *H. pylori *in gastric biopsy specimens. In their study the authors concluded that nested PCR amplification assays are necessary to detect *H. pylori*-DNA in gastric biopsy specimens. From this study we conclude that MDA-amplified DNA derived from minute amounts of biopsy specimen DNA is well suited for PCR-amplification and subsequent sequencing of M13 sequence-tagged amplicons. Thus, it is possible to avoid nested PCR amplification assays which often have to be combined with Southern blot analysis to increase the sensitivity and specificity [28,30].

## Conclusion

The highly sensitive PCR-based molecular typing methods described here enables reliable and concurrent genotyping analyses of bacterial and host cellular DNA from the same biopsy specimen at a reasonable cost and time. Although the power of this approach was demonstrated with concurrent *H. pylori *and cytokine SNP analysis, the principles and technologies could be applied to any situation in which microbial populations are analysed, and in particular for addressing questions concerning microbial-host interactions associated with human health in general.

## Competing interests

The authors declare that they have no competing interests.

## Authors' contributions

AR, KB Y-QS and HJM participated in the conception, design, drafting of the manuscript, and final approval of the version to be published. AR and HJM were responsible for the acquisition, analysis and interpretation of data. KB selected the biopsy specimens in the study. All authors have read and approved the final manuscript.
